# Operative management of a subtrochanteric fracture in severe osteoporosis. *a case report*

**DOI:** 10.1186/1757-1626-1-193

**Published:** 2008-09-30

**Authors:** Shabir Ahmed Dhar, Manzoor Ahmed Halwai, Mohammed Iqbal Wani, Mohammed Farooq Butt

**Affiliations:** 1 Department of Orthopaedics, Government Medical College, Srinagar, Jammu and Kashmir, India

## Abstract

**Background:**

Fractures of the subtrochanteric region of the femur provide several challenges to the operating surgeon due to anatomic and biomechanical peculiarities inherent to this region. These challenges are compounded several times in a severely porotic bone.

**Case presentation:**

We report a case with severe osteoporosis who sustained a subtrochanteric fracture and was managed with a Dynamic condylar screw DCS. Three years after the surgery the patient is pain free and has a full range of motion.

**Conclusion:**

This highlights the fact that the DCS provides a viable alternative in the management of fractures of the subtrochanteric region in severe osteoporosis. This advantage is particularly manifest in settings where the image intensifier is not easily available.

## Background

The existence of osteoporosis has been documented in Egyptians as far back as 990 B.C. [1]. The incidence of fragility fractures around the hip is rising exponentially. [2] These fractures account for a significant economic strain on the health care system of a country. [3]

Subtrochanteric fractures pose significant challenges for fixation in terms of anatomic and biomechanical reasons. These include a very high stress across the medial cortex [1200 lb/sq inch], smaller cross sectional area at the isthmus and the occurrence of shear across the fracture. These challenges are further compounded by the presence of powerful muscle vectors. [4]

The major problem in fractures of the osteoporotic bone is fixation of the device to the bone as bone failure is commoner than implant breakage.

We report a case with subtrochanteric fracture of the femur with Singh's grade one osteoporosis which was managed by fixation with a dynamic condylar screw. At three years follow-up the patient had a normal range of motion and was pain free.

## Case presentation

An 87 year old woman patient reported to the outdoor department of our hospital with a history of having sustained a fall in her bathroom. The patient complained of tenderness and pain in the right hip and thigh. The extremity was externally rotated and shortened. Movement elicited pain in the area of the right hip. Radiographs of the right femur revealed a transverse fracture of the right subtrochanteric region. On assessing the trabecular pattern of the trochanteric region the patient was found to have Singh's grade one porosis with near absence of the trabeculae in the femoral head and neck.

The patient was put on traction and advised to undergo surgery in view of the nature of the fracture and the requirement of early ambulation. Dual X-ray absorbsiometry showed a T score greater than 2.5 standard deviations below normal.

In view of the extremely thin cortices of the femur, it was felt that the oft required schanz pin assisted reduction, needed for guide wire insertion in interlocking nailing, could damage the cortices at the insertion site. Besides this the possibility of cut out precluded the use of dynamic hip screw fixation. The DCS along with cancellous screws was chosen to circumvent these problems.

The fracture was reduced on a traction table after opening the area. Fixation was held with a dynamic condylar screw [DCS] and 95 degree barrel plate. In view of the tenuous nature of the cortices the plate was affixed with four cancellous screws on either side of the fracture.

Post operatively the patient underwent supervised physiotherapy over a period of six weeks. At ten weeks the fracture had united and the patient was allowed full weight bearing. The patient simultaneously was put on treatment with bisphosphonates for the underlying osteoporosis which was primarily of the senile variety.

At the last follow-up [3 years post operative] the patient was pain free with full range of motion of the hip joint. The patient's cortex thickness had also improved at this time as seen on radiographs. The patient was advised as to the potential complications of removal of hardware after which she chose to avoid removal of the implant.

## Discussion

In planning treatment in older patients with fractures of the osteoporotic bone, several important factors are to be considered. The functional demands of the elderly are different from young healthy and long term immobilization in bed must be avoided. Delaying treatment has been reported to increase mortality. [5]

Reduced bone mass, increased bone brittleness and medullary expansion must be factored in when deciding the type of surgical method to be used.

Improved implant design and surgical techniques are in a constant race to keep pace with increasing demands for stable fixation of these fractures.

Immobilization in splints and casts causes further immobilization resulting in stiffness and worsening the porosis. A cast also does not control fracture shortening which is often seen in osteoporotic bone.

External fixators can result in pin loosening, infection, resorption which may manifest as further fractures.[6]

Cement can be used to augment fixation but can sometimes interfere with fracture healing.

If plates are used, they should be used as tension bands which require cortical contact opposite the plates. In addition, long plates should be used as they will distribute the forces over a larger area reducing the risk of bone failure. [7]

Intramedullary nails provide several advantages in such fractures but the requirement of expensive image intensifiers often restricts the use of this equipment.

The costs and outcome of hip fractures are often closely monitored. The mortality attributable to osteoporosis is most obviously associated with hip fractures with the highest incidence occurring in the first six months after the fracture.

Various implants have been used for fracture fixation in the upper femur. These include ender nails, short femoral nails, sliding hip screws, fixed nail plate, Kuntscher Y nail and percutaneous compression plate. Many series report complications with these methods. However none of these series exclusively focus on cases with severe ostemalacia with cortices as thin as found in our patient. Among the most common treatments for extracapsular fractures, the average rate of cutout was 2.6% in patients receiving a short femoral nail, 3.1% in those receiving a sliding hip screw, 3.6% in those receiving a Medoff sliding plate and 6.7% in those receiving an Ender nail. Operative fractures of the femur occurred in 2.4% of those receiving Ender and in 2.7% in short femoral nails. The rate of operative fractures was negligible in those receiving a sliding hip screw (0.4%). The overall average rate of valgus deformity was 7.7% and that of leg shortening 9.4%.[8] A study conducted by Yoshmine et al on bones with grade 1–3 porosis showed a cut out rate of 36%.[9]

This is the first case report to our knowledge, which describes the use of the DCS along with cancellous screws in the management of the subtrochanteric fracture in a bone with osteoporosis of such severity. The DCS has the advantage of giving a good hold in the femoral neck area by virtue of its position while avoiding complications of pull out. Cancellous screws were used to improve the hold further.

The purpose of reporting this case was to highlight the possibility of using the DCS in a financially constrained setting in such cases.

## Conclusion

The DCS provides a good alternative to intramedullary nails in the fixation of subtrochanteric fracture in the severely osteoporotic bone. This advantage is particularly manifest in resource constrained settings where image intensifiers may not be available.

## Abbreviations

DCS: Dynamic Condylar Screw.

## Competing interests

All authors confirm that there are no conflicts of interests, including financial and personal relationships with other people, or organisations, that could inappropriately influence (bias) their work.

## Authors' contributions

All authors were involved in patient management or writing of the manuscript.

## Consent

Written informed consent was obtained from the patient for publication of this case report and accompanying images.

**Figure 1:**
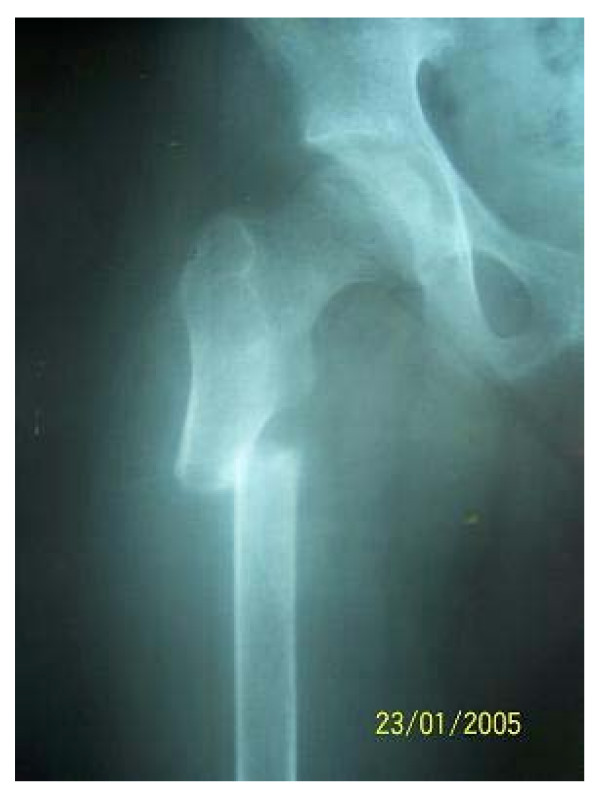
Showing the subtrochanteric fracture in the porotic bone.

**Figure 2:**
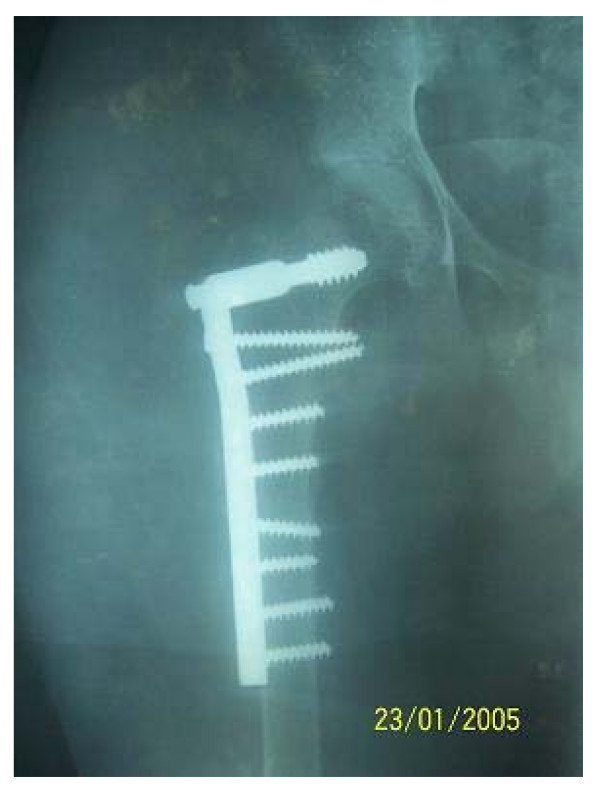
Showing fixation with a dynamic condylar screw and plate and cancellous screws.

**Figure 3:**
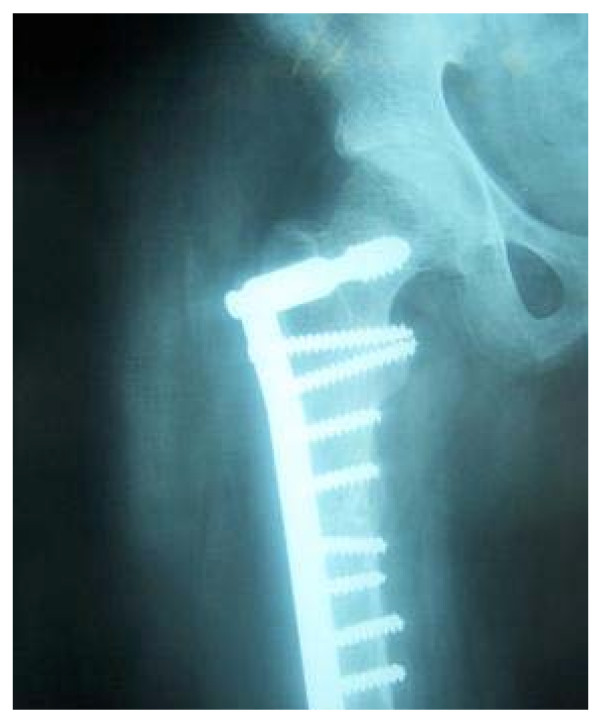
Showing the fracture status at three years follow up.
